# The Effect of Arsenic Mitigation Interventions on Disease Burden in Bangladesh

**DOI:** 10.1289/ehp.6866

**Published:** 2004-06-17

**Authors:** Kamalini M. Lokuge, Wayne Smith, Bruce Caldwell, Keith Dear, Abul H. Milton

**Affiliations:** ^1^Australian National University, National Centre for Epidemiology and Population Health, Acton, Australian Capital Territory, Australia; ^2^University of Newcastle, Centre for Clinical Epidemiology and Biostatistics, Newcastle, New South Wales, Australia

**Keywords:** arsenic/adverse effects, Bangladesh, burden of disease, diarrhea, risk assessment, water pollutants, water supply

## Abstract

Many interventions have been advocated to mitigate the impact of arsenic contamination of drinking water in Bangladesh. However, there are few data on the true magnitude of arsenic-related disease in Bangladesh nationally. There has also been little consideration given to possible adverse effects of such interventions, in particular, diarrheal disease. The purpose of this study was to estimate and compare the likely impacts of arsenic mitigation interventions on both arsenic-related disease and water-borne infectious disease. We found that arsenic-related disease currently results in 9,136 deaths per year and 174,174 disability-adjusted life years (DALYs; undiscounted) lost per year in those exposed to arsenic concentrations > 50 μg/L. This constitutes 0.3% of the total disease burden in Bangladesh in terms of undiscounted DALYs. We found intervention to be of overall benefit in reducing disease burden in most scenarios examined, but the concomitant increase in water-related infectious disease significantly reduced the potential benefits gained from intervention. A minimum reduction in arsenic-related DALYs of 77% was necessary before intervention achieved any reduction in net disease burden. This is assuming that interventions were provided to those exposed to > 50 μg/L and would concomitantly result in a 20% increase in water-related infectious disease in those without access to adequate sanitation. Intervention appears to be justified for those populations exposed to high levels of arsenic, but it must be based on exposure levels and on the effectiveness of interventions not only in reducing arsenic but in minimizing risk of water-related infections.

There has been growing concern regarding the widespread exposure of the Bangladeshi population to arsenic in tube-well water (Smith et al. 2000). Many interventions and alternative water sources have been advocated on the grounds that they are effective in reducing arsenic ingestion. However, limited consideration has been given to possible adverse effects of such interventions, in particular, water-related infectious diseases such as diarrhea. Although this issue has been raised (Caldwell 2003; MacDonald 2001), there has been no evaluation published allowing a meaningful assessment of the competing risks involved in mitigation. Such an assessment is required before the development of effective policy recommendations for arsenic mitigation in Bangladesh.

In this article, we present an evaluation of the possible change in overall burden of disease resulting from implementation of arsenic mitigation interventions in Bangladesh and compare likely impacts on both arsenic-related disease and water-borne infectious disease.

## Materials and Methods

Arsenic-related disease due to chronic exposure through drinking water has a relatively low incidence and a latency of up to decades for most end points significant to a burden of disease assessment (National Research Council 2001). However, case fatality rates for arsenic-exposure–related sequelae such as internal cancer are high, particularly in a country such as Bangladesh where access to health care is limited. In contrast, diarrheal and other water-related infectious diseases, although having a comparatively low case fatality rate, have a much higher incidence. Additionally, 90% of the disease burden due to diarrhea occurs in children younger than 5 years of age (Pruss et al. 2002), unlike arsenic, which affects primarily older adults. To provide a basis for comparing end points with such diversity in the population profile, we calculated mortality rates and disability-adjusted life years (DALYs) lost for end points related to these two risk factors.

DALYs are the measure used by the Global Burden of Disease (GBD) study to assess and compare burden of disease due to varied risk factors and end points. The DALY is a summary health measure that accounts for mortality at different ages and for both the severity and duration of morbidity (Murray et al. 2002). By using DALYs, a comparison can also be made between the impacts of arsenic, of water-related infection, and of arsenic mitigation interventions overall, with other causes of disease in Bangladesh evaluated by the GBD study. DALYs, which measure loss of healthy life, are useful for assessing the impact of interventions and comparing predicted health states (Murray and Acharya 1997); therefore, we chose DALYs over other measures of quality-adjusted life. Because one purpose of the present study was to provide input into policies for arsenic mitigation, we include estimates discounted at 3% (the discounting rate used in the standard GBD figures) alongside those with zero discounting.

The disease burden attributable to each risk factor was estimated using cause-specific rates of mortality and DALYs for the Southeast Asia region (SEAR), D subregion, for the year 2001 published in the 2002 *World Health Report* [World Health Organization (WHO) 2002]. These estimates were regarded as the most appropriate for Bangladesh because the SEAR-D subregion includes those countries within the SEAR that have high child and adult mortality rates (India, Bangladesh, and Pakistan).

To calculate disease burden for those end points not disaggregated in the GBD study, we obtained background mortality rates applicable to the Bangladeshi population from the literature. These data were then entered into the formulas for DALYs given in the GBD study (Murray and Lopez 1996). The rates of disease and assumptions used for each specific exposure and sequelae are defined below.

We obtained demographic data from multiple sources. Total population by *thana*-level administrative unit was obtained from the Bangladesh Bureau of Statistics and derived from the 1991 national census (Bangladesh Bureau of Statistics 2002). The age structure of the Bangladeshi population was obtained from the 1999–2000 Bangladesh Demographic and Health Survey (BDHS) (National Institute of Population Research and Training 2001).

Relative risk estimates from published literature were entered into the formulas for attributable fraction given by Rockhill et al. (1998). The calculated exposure and disease-specific attributable fractions were then applied to relevant background estimates to obtain the total disease burden due to the factors under study.

### Disease burden from arsenic exposure.

We calculated estimates of the arsenic-exposed population by different age strata by assuming that *a*) the exposed group had an overall similar age structure to the population surveyed in the 1999–2000 BDHS (National Institute of Population Research and Training 2001); *b*) total population numbers within each *thana* subunit were similar to those of the 1991 national census; and *c*) exposure was through use of water from shallow tube wells. Population estimates were adjusted for levels of shallow tube-well use, currently estimated at 87% (Caldwell 2003).

Data on arsenic levels in tube wells used in this study were obtained from a British Geological Survey (BGS) survey of tube wells in Bangladesh (Kinniburgh and Smedley 2001). This survey is the only one currently published that provides nationally representative data. Table 1 shows the average and median arsenic concentrations for various ranges calculated using these data.

We calculated the population exposed to arsenic at different levels by using the distribution of arsenic exposure estimated from the BGS data (Kinniburgh and Smedley 2001). and applying it to 1991 national census data (Bangladesh Bureau of Statistics 2002). This was done for each *thana*, the smallest administrative subunit for which data were available. The calculated proportions of the population in each exposure strata are shown in Table 2.

### Arsenic-related end points.

The quality of studies detailing associations between health outcomes and arsenic exposure varies. The literature has been reviewed by expert committees from the U.S. Environmental Protection Agency (EPA) Science Advisory Board (U.S. EPA 2001a), in the 2001 United Nations Synthesis Report on Arsenic in Drinking Water (Abernathy 2001), and by the American Council on Science and Health (Brown and Ross 2002). Table 3 stratifies the level of evidence for a possible association between arsenic and these disease end points according to these key reviews. From those end points that any of these organizations considers to have strong or reasonably strong evidence for an association, we selected all that directly contribute to disease burden. We included lung, bladder, kidney, and skin cancers; ischemic heart disease; and diabetes mellitus.

### End points not included.

Skin alterations are the most common manifestation of chronic arsenic exposure. However, as in the GBD study, the nonmalignant manifestations were assumed to cause minimal disability and no independent increase in mortality; therefore, we did not include nonmalignant skin alterations as end points in our study.

Peripheral vascular disease (PVD) has been noted in arsenic-exposed populations worldwide, but there is continuing debate over the association between arsenic and the more severe forms of PVD, in particular, the role of other chemicals in the causation of blackfoot disease (Yang et al. 2002). For this reason, and because it has not been noted in Bangladesh, PVDs such as blackfoot disease were not included.

Although hypertension has been found to be associated with arsenic exposure (Rahman et al. 1999), it is not a contributor to disease burden of itself, but a risk factor for end points that have been included. Hypertension was therefore not included as a separate end point.

### Calculating arsenic-related attributable fraction of disease.

Although there are some studies on arsenic-related disease in Bangladesh, none provide reliable population-level estimates of risk. Despite limitations, and uncertainty regarding the level of exposure, the data we used were from studies carried out in Taiwanese populations (Tsai et al. 1999). We used these data because they are still recognized as the most reliable source of dose–response information on exposure to arsenic in drinking water currently available (National Research Council 2001; U.S. EPA 2001b).

### Cancers associated with arsenic.

Relative risks for lung, liver, bladder, and kidney cancer were published in a review by Smith et al. (1992). A major issue related to the use of these estimates was the wide range within each category of exposure. In particular, the lowest exposure group covered the range from 0 to 300 μg/L [weighted average concentration, 170 μg/L (U.S. EPA 1988)]. However, the average concentration in tube wells in Bangladesh for this range is much lower: 170 μg/L is closer to the average concentration in tube wells in Bangladesh in a range of 100–300 μg/L and not 0–300 μg/L (Table 1). Most studies also suggest that the threshold for internal malignancies related to arsenic exposure is > 100 μg/L (Chiou et al. 2001; Guo 2000). The risks for the Taiwanese population exposed at an average concentration of 170 μg/L are therefore probably most applicable to the population in Bangladesh exposed within the 100–300 μg/L range. To account for this, we calculated a range of attributable fractions, each using the same relative risk for the lowest exposure category, but with the proportion of the population exposed to that risk level varied to the proportion in Bangladesh exposed at 0–30, 10–300, 50–300, or 100–300 μg/L.

We applied the relative risks due to arsenic exposure obtained from these Taiwanese data to estimates of DALYs and deaths for each end point in the GBD SEAR-D subregion (WHO 2002). However, the GBD study does not provide disaggregated data for either kidney or nonmelanotic skin cancer. In the case of kidney cancer, we calculated mortality using age-specific background cancer mortality rates in Bangladesh published by the International Agency for Research on Cancer (IARC; Ferlay et al. 2001). For Bangladesh, where cancer registry data are not available, mortality was estimated from Indian registry incidence data using country/regional survival (Parkin 1986). However, the background rate we used for kidney cancer is actually an aggregate of kidney cancer along with cancers of other urinary organs and therefore will overestimate the risk of kidney cancer alone.

There are no reliable estimates for either incidence or case fatality rates for nonmelanotic skin cancers applicable to Bangladesh. Arsenic is not associated with melanoma, which dominates the estimates from the combined skin cancer categories routinely reported, including by IARC; therefore these rates could not be used. By combining calculated lifetime excess risk of skin cancer due to arsenic exposure in Bangladesh (Khan et al. 2003) with a case fatality rate of 14.3% for arsenic-related skin cancers over a 5-year period (Yeh 1973), it was possible to derive an estimate of the number of skin cancer deaths per year due to arsenic exposure in Bangladesh.

For both skin cancer and kidney disease, lack of data meant that only total mortality and years of life lost (YLL), a subcomponent of DALYs, could be calculated.

### Noncancer effects associated with arsenic.

Several studies have noted an association between arsenic exposure and cardiovascular disease in populations from Taiwan, Chile, and the United States, and an increased prevalence of hypertension has been noted in populations exposed to arsenic, including in Bangladesh (Rahman et al. 1999). Three possible sources of risk estimates were a cohort study by Chen et al. (1996), an ecologic level study by Tsai et al. (1999), and a study in Bangladesh on prevalence of hypertension (Rahman et al. 1999). Chen et al. (1996) were unable to provide precise estimates of risk at levels of exposure < 500 μg/L. We did not use the Rahman et al. (1999) study because exposure was not directly determined but inferred from the presence of arsenic-related skin lesions. The relative risk of death from ischemic heart disease in arsenic-exposed compared with nonexposed populations was therefore obtained from Tsai et al. (1999).

Diabetes has also been associated with arsenic exposure in some studies, including one conducted in arsenic-exposed populations in Bangladesh (Rahman et al. 1998), but again, this study suffered from the same limitations of exposure measurement. The ecologic study by Tsai et al. (1999) on the Taiwanese population found standardized mortality ratios (SMRs) for death from diabetes of 1.35 for women and 1.55 for men in a population exposed to elevated arsenic levels in drinking water compared with a local reference population; we used these figures to calculate attributable risk.

### Water-related infectious diseases.

There are several infectious diseases that are water related, including infective causes of acute diarrhea, helminth infections, schistosomiasis, and water-washed diseases such as trachoma (Esrey et al. 1991). Within the SEAR-D subregion as a whole, > 99% of the disease burden from these infections is attributable to diarrheal disease. Pruss et al. (2002) estimated that the global disease burden due to diarrhea and other water-related infectious diseases attributable to water, sanitation, and hygiene is 4.0% of all deaths and 5.7% of the total burden of disease in DALYs lost. Proportionately, diarrheal disease is an even greater contributor to the burden of disease in developing countries such as Bangladesh (Hussain et al. 1999). Considering that the overall burden of diarrheal disease is so high, it is important to evaluate the possible impacts that currently recommended changes in water supply aimed at arsenic mitigation may have on water-related infectious disease, in particular, diarrheal disease.

### Attributable risk due to change in water supply.

In the context of Bangladesh, the most appropriate technology in terms of microbiologically clean water was and is tube wells. To assess the possible additional burden of disease resulting from changes to current arsenic-contaminated water supplies, it is necessary to estimate the magnitude of this effect.

A recent study into the global burden of water-related illness disease categorized exposure to diarrheal disease from water supply into several levels (Pruss et al. 2002). The Global Water Supply and Sanitation Assessment classification of water supplies and sanitation infrastructure was the source of the definitions used by Pruss et al. (2002) to categorize the exposure level of subgroups of the world population in terms of access to water and sanitation services [WHO and the U.N. Children’s Fund (WHO/UNICEF) 2002]. Levels of relevance to this assessment and the risk reduction when moving between these levels for the present study are presented in Table 4.

A recent WHO/UNICEF joint report (WHO/UNICEF 2001) used serial surveys of coverage to estimate current levels of access to water supply and sanitation in Bangladesh. Access to improved sanitation in rural areas is 41%; therefore, 59% of the population has inadequate access to these services (WHO/UNICEF 2001).

The current status of most of the Bangladeshi population would primarily be within the Vb level of risk (improved water supply but no improved sanitation; Table 4). In evaluating possible arsenic mitigation options, the feasible alternatives include various forms of surface water, treatment of tube-well water before consumption, and the use of available uncontaminated tube wells. All involve a possible change in either the quality or quantity of water available to the household for use.

A transition to surface water sources such as unimproved dug wells, ponds, or streams would mean a change in exposure status from level Vb to level VI (no improved water supply and no sanitation) and therefore an estimated increase in diarrheal disease of 20%. The data presented below are based on the assumption of the equivalent of this change in risk for arsenic mitigation interventions for those in the subpopulation without access to improved sanitation.

### Mortality due to diarrheal disease.

Although several studies in Bangladesh have examined diarrheal incidence in childhood, relatively few have assessed mortality. Because reliable estimates require large sample sizes, most studies evaluating diarrheal mortality do so in the context of hospital-based case studies and cannot provide estimates of overall mortality in the community.

One extensive source of data on Bangladesh comes from studies conducted in the long-term follow-up area of the International Center for Diarrhoeal Disease Research, Bangladesh (ICDDR-B; Fauveau 1994). However, this population has been studied intensively over many decades and may not therefore be representative of the general population. In the present study, we used the GBD SEAR-D water-related infectious disease morbidity rates (WHO 2002), which are conservative relative to ICDDR-B estimates.

## Results

Table 1 gives the average and median arsenic concentrations for various ranges of exposure, calculated using BGS tube-well survey data from Bangladesh (Kinniburgh and Smedley 2001).

Table 2 details the proportion of population estimated to be within each exposure range in those regions of Bangladesh surveyed by the BGS (Kinniburgh and Smedley 2001).

Table 5 provides results of the estimated total burden of disease due to exposure to arsenic at concentrations > 50 μg/L.

Projections of intervention impact on water-related infectious disease are shown in Table 6, given that 59% of the population in Bangladesh does not have access to improved sanitation and assuming that interventions are used by all those exposed to > 10 μg/L (scenario A) or all those exposed to > 50 μg/L (scenario B).

Projections of the net change in disease burden as a result of intervention are shown in Table 7. These projections assume a 100% reduction in arsenic-related disease, along with a 20% increase in water-related infectious disease in the subgroup without access to sanitation. Also included is an estimate of the net effect with different thresholds for the effects of arsenic on lung, bladder, and kidney cancer. Table 8 presents the predicted increase in infectious disease burden as a percentage of current total arsenic-related disease burden and is therefore the minimum reduction in current arsenic-related disease burden necessary to achieve any net decrease in overall disease burden through intervention.

Table 9 presents the arsenic-related burden of disease in those exposed to concentrations > 50 μg/L as a proportion of total burden of disease in Bangladesh and as a proportion of the disease burden due to other selected causes.

## Discussion

Arsenic contamination of drinking water is a major health issue for those living in affected areas of Bangladesh. However, the present study demonstrates that much of the benefit obtained from intervention may be negated by a concomitant increase in water-related infectious disease. Currently, in the evaluation of arsenic mitigation interventions, the emphasis is on assessing their impact on arsenic levels. Clearly this is important because inefficient interventions are likely to have little overall benefit and may even have adverse net impacts. However, all suggested mitigation interventions must be considered not only from the perspective of reducing arsenic-related morbidity and mortality but also from the overall health perspective.

There are a number of methodologic issues that are important in considering the results of the present study: *a*) estimates of exposure; *b*) estimates of disease burden from arsenic exposure, in particular the end points chosen for inclusion; *c*) extrapolation of risk estimates of arsenic exposure from different populations, with differing exposure levels; *d*) estimates of the effectiveness of arsenic mitigation interventions; *e*) estimates of disease burden from diarrhea; and *f*) estimates of diarrheal risk from arsenic mitigation interventions, including assumptions about changing from improved to unimproved water supplies.

### Estimates of exposure.

Exposure data were obtained from the BGS survey of tube wells (Kinniburgh and Smedley 2001), the only nationally representative data on tube-well contamination currently available. However, this survey sampled fewer than 4,000 tube wells; therefore, at the *thana* level, exposure was inferred from relatively few data points. These estimates can be refined only if more comprehensive tube-well surveys, using nationally representative sampling frames, are conducted.

### Estimates of disease burden from arsenic exposure.

We took an inclusive approach in estimating the disease burden from arsenic exposure. The GBD rates for cardiovascular disease and diabetes may be an overestimate for Bangladesh because members of this primarily poor and rural population are likely to have lower cardiovascular disease and diabetic mortality rates than those in the urban Indian populations, whose levels are more likely to match the SEAR-D estimates (WHO 2002). The disease burden due to these end points is therefore likely to be biased toward a beneficial effect of arsenic mitigation. The burden of disease estimates for arsenic are dominated by the contribution of cardiovascular disease, but the association between arsenic exposure and cardiovascular disease remains ambiguous, at least in strength of association. Because the strength of this association will determine whether interventions are likely to cause good or harm, it is crucial that valid estimates of this association are available, particularly at lower arsenic exposure levels.

End points resulting from chronic arsenic exposure chosen for inclusion were those that major reviews considered to have strong or reasonably strong evidence of a causal link. Numerous other end points have been found to be associated with arsenic exposure. However, the evidence is much less definitive, in terms of both whether an association exists and its strength. Additionally, data do not currently exist to allow a meaningful estimate of the burden of disease resulting from such end points. However, excluding them from this study may slightly underestimate arsenic-related burden of disease.

### Extrapolation of risk estimates of arsenic exposure from different populations with differing exposure levels.

For all arsenic-related end points, we derived risk estimates from a different population to the study population, assuming the same exposure–risk relationship. The source Taiwanese population is described as being largely rural, engaged in farming, fishing, and salt production, of below average socioeconomic standard, and with a low-protein diet based primarily on rice and sweet potatoes (Wu et al. 1989). In terms of these factors, the current Bangladeshi population is fairly similar to the Taiwanese population of 40 years ago from which the data are derived (Bangladesh Bureau of Statistics 2002). The primary caloric source is rice, and malnutrition levels are high (Ahmed 1992). For these reasons, we made no adjustment for fluid intake and body mass, as has been done when extrapolating Taiwanese data to the U.S. population (Morales et al. 2000). However, the risks for the Taiwanese population exposed at an average concentration of 170 μg/L (range, 0–300 μg/L) are probably most applicable to the population in Bangladesh exposed within the 100–300 μg/L range and not the 0–300 μg/L range (discussed in “Materials and Methods”).

### Estimates of the effectiveness of arsenic mitigation interventions.

Arsenic mitigation interventions, if given to those exposed to > 50 μg/L, would need to achieve at least a 77% reduction in arsenic-related disease burden to result in a net reduction in DALYs. Arsenic mitigation interventions cannot achieve a 100% reduction in disease burden for several reasons, and even reductions of 70–80% are doubtful. It is unlikely that any of the interventions widely accessible in Bangladesh would be 100% effective, due to both compliance and efficacy, and the degree to which arsenic contamination of irrigation water and resultant intake through food contributes to disease burden is unclear.

Therefore, assuming a 100% reduction in arsenic-related disease after intervention, as was done for all estimates in this study, is likely to bias results toward a beneficial outcome from intervention.

### Estimates of disease burden from diarrhea.

The GBD study rates (WHO 2002) used in the estimations are lower than those from recent studies in Bangladesh on the disease burden from diarrhea; Streatfield et al. (2001) estimated the disease burden attributable to diarrheal disease in Bangladesh as 11% of all deaths and 12.1% of DALYs. The SEAR-D rates used (WHO 2002) were 6.2% of deaths and 7.2% of undiscounted DALYs, which are both almost half that of Streatfield et al. (2001). The background rates of diarrheal disease used are therefore conservative in the context of Bangladesh. This is again likely to bias results toward an overall beneficial effect of arsenic mitigation.

### Estimates of diarrheal risk from arsenic mitigation interventions.

The association between incidence of diarrheal disease and water supply was categorized into several levels by Pruss et al. (2002). Based on these data, there is a 20.8% increase in risk when moving between level Vb (improved water supply but no basic sanitation) to level VI (no improved water supply and no basic sanitation). Studies conducted in developing countries including Bangladesh found that water and sanitation interventions have a proportionately greater impact on child mortality as opposed to morbidity [a 26% reduction in morbidity, compared with a 55% reduction in overall mortality, and a 65% reduction in diarrhea related mortality (Esrey et al. 1991)]. Because the attributable risks used applied to changes in morbidity, it is likely that we underestimated impacts on mortality.

It is clearly appropriate to assume an increased risk when the intervention involves moving from tube wells to dug wells that are not sanitary protected (constructed to be relatively protected from microbial contamination), or moving to other forms of surface water that are unimproved. However, a change from contaminated water to uncontaminated tube wells would, at face value, appear to involve no change in exposure status. Assuming any individual household would prefer to use the most convenient well, usually the closest and often within the household compound, any change in the tube well would presumably involve a change to an uncontaminated but less convenient tube well, in terms of either distance or the number of individuals using the well for water. Aside from compliance issues, this also increases the risk of water-borne disease. Studies have found that in terms of protection against infectious disease, the quantity of water used is as important or even more important than the quality of water used (Esrey et al. 1991), and that the quantity of water used is directly related to the distance to the water source and the number of users (Hoque et al. 1989). Thus, even a change in the tube well used may increase the risk of diarrheal disease.

There is also evidence to suggest that arsenic filtration systems may increase the risk of water-related infections. The main risk of filter systems is through increased handling and storage of water within the household, and past studies have shown that household storage and handling is a significant source of contamination, perhaps the major source (Molbak et al. 1989). This assumption is supported by a field study evaluating arsenic removal systems in Bangladesh, which found that such systems had higher levels of microbial contamination in the filtrated water than in the tube wells from which the water was taken (Sutherland et al. 2002) and may therefore increase the risk of water-related infectious disease.

### Latency.

Any impact that changes in water supply have on incidence of arsenic-related disease will be delayed, probably for several years. Estimates of the latency period for arsenic-related chronic disease vary greatly, but most are in the range of several decades. For bladder, lung, and liver cancer, estimates range to > 40 years (Chen et al. 1986). However, the impact of arsenic mitigation interventions on diarrheal disease will be immediate. Because maximal arsenic-related reductions would be delayed for a number of years, there would be an overall increase in mortality in the period immediately after initiation of any intervention. Because the results given here apply only once equilibrium has been reached, they do not take into account this period and therefore, again, are biased toward a beneficial effect of mitigation.

## Conclusions

There are many areas where limited data affected the validity of the estimates obtained in this study, including lack of data on the long-term effects of arsenic exposure at the lower ranges; lack of reliable population-level estimates of risk related to arsenic exposure in Bangladesh, particularly for those more common end points such as cardiovascular disease that are likely to constitute the bulk of disease burden; and imprecise data on exposure nationally.

At present, there are inadequate data to reliably meet these needs, and formulating policy options before the availability of such data carries potentially significant risks. The present study is an attempt to make a quantitative assessment of the impacts of intervention. As data become available in those areas where it is currently lacking, further refinements will allow more precise estimates of benefit and risk.

As Table 9 demonstrates, arsenic-related disease resulting from exposure to arsenic concentrations > 50 μg/L constitutes 0.3% of the total disease burden in Bangladesh in terms of undiscounted DALYs, and although it is a significant cause of disease burden in exposed groups, nationally it is of less importance than many other risk factors.

Interventions must be used effectively in a country such as Bangladesh, where resources are limited and multiple competing interests exist. In the case of arsenic mitigation, this means ensuring that interventions are targeted to those areas where exposure has been confirmed, and that those interventions provided achieve significant reductions in arsenic exposure without concomitantly causing substantial increases in other risks such as water-related infectious disease.

As these estimates demonstrate, the effects of arsenic mitigation are double-edged, and intervention appears to be clearly justifiable at present only within the higher levels of exposure. There is an urgent need for studies evaluating alternative water sources in terms of not only their effectiveness in reducing arsenic intake but also their associated effect on water-related infections.

## Figures and Tables

**Table 1:** Arsenic concentrations in tube wells in Bangladesh.

Concentration range (μg/L)	Average concentration within range (μg/L)	Median concentration within range (μg/L)
0–300	33	< 10
10–300	82	56
50–300	132	108
100–300	180	170
> 300–600	421	406
> 10–500	107	63
> 500	628	572
> 50–500	167	130
> 600	755	668

Calculated using data from (Kinniburgh and Smedley (2001).

**Table 2:** Distribution of arsenic exposure across the population of Bangladesh.

Arsenic concentration range (μg/L)	Percentage of population exposed to drinking water contaminated at this level
≤10	58.8
> 10–50	16.4
> 50–100	8.6
> 100–300	10.9
> 300–600	4.5
> 600	0.83

Calculated using BGS tube-well survey data (Kinniburgh and Smedley 2001) and population data from the 1991 Bangladesh national census (Bangladesh Bureau of Statistics 2002).

**Table 3:** Strength of evidence for a causal link between arsenic and various end points.

Reference	Level of evidence	Exposure-related disease end point
U.S. EPA 2001a	Strong	Lung, bladder cancer
	Reasonably strong	Ischemic heart disease, diabetes mellitus, hypertension, skin cancer
	Suggestive	Prostate cancer, nephritis and nephrosis, hypertensive heart disease, nonmalignant respiratory disease
Abernathy 2001	Strong	Skin, lungs, bladder, kidney cancer, skin hyperkeratosis and pigmentation changes
	Reasonably strong	Hypertension, cardiovascular disease
	Suggestive	Diabetes, reproductive diseases
	Weakest	Cerebrovascular disease, long-term neurologic effects, cancer at sites other than skin, lung, bladder, and kidney
Brown and Ross 2002	May cause	Skin, lung, bladder cancer, cutaneous effects
	Possible	Kidney, liver, prostate, and other cancers
	Some evidence	Cardiovascular/cerebrovascular diabetes, reproductive diseases

**Table 4:** Change in risk of diarrheal disease due to improvements in water supply and sanitation services.

Level	Description of level	Risk difference
VI	No improved water supply and no basic sanitation in a country that is not extensively covered by those services, and where water supply is not routinely controlled	Index
Vb	Improved water supply and no basic sanitation in a country that is not extensively covered by those services, and where water supply is not routinely controlled	20.8%
Va	Basic sanitation but no improved water supply in a country that is not extensively covered by those services, and where water supply is not routinely controlled	37.5%
IV	Improved water supply and basic sanitation in a country that is not extensively covered by those services, and where water supply is not routinely controlled	37.5%

Data from (Pruss et al. (2002)

**Table 5:** Burden of disease incurred in Bangladesh each year due to arsenic levels > 50 μg/L.

		DALYs	
Disease	Deaths	Undiscounted	Discounted at 3%
Diabetes mellitus	351	10,524	7,628
Ischemic heart disease	5,128	91,616	67,380
Tracheal, bronchial, lung cancers	2,100	39,759	28,921
Bladder cancer	1,346	25,432	17,121
Kidney cancer*^a^*	85	3,463	1,840
Skin cancer*^a^*	126	3,379	2,120
Total disease burden	9,136	174,174	125,010

a Includes only YLL and not years lived with disability.

**Table 6:** Estimated increase in water-related infectious disease burden caused by arsenic mitigation.

		DALYs	
Scenario	Deaths	Undiscounted	Discounted at 3%
A: Assuming interventions were used by all those exposed to arsenic > 10 μg/L	3,370	218,198	97,659
B: Assuming interventions were used by all those exposed to arsenic > 50 μg/L	2,080	134,671	60,275

**Table 7:** Net impact of arsenic mitigation on burden of disease in Bangladesh.

			DALYs	
Population supplied with intervention	Threshold for arsenic-related lung, bladder, and kidney cancer	Deaths	Undiscounted	Discounted at 3%
All those exposed to arsenic levels > 10 μg/L	No threshold	6,623	−27,251	39,173
	> 50 μg/L	5,765	−44,024	27,351
	> 100 μg/L	5,072	−58,785	17,324
All those exposed to arsenic levels > 50 μg/L	No threshold	7,055	39,503	64,735
	> 100 μg/L	6,362	24,741	54,707

A negative number signifies a net overall increase in DALYs lost.

**Table 8:** Predicted increase in infectious disease burden resulting from arsenic mitigation, given as a percentage of the disease burden currently incurred through arsenic exposure.*^a^*

		DALYs (%)	
Population supplied with intervention	Deaths (%)	Undiscounted	Discounted at 3%
Exposed to arsenic levels > 10 μg/L	34	114*^b^*	71
Exposed to arsenic levels > 50 μg/L	23	77	48

a Assuming no threshold for arsenic-related disease.

b Percentage is > 100 because the total arsenic-related burden of disease that can be removed through mitigation is less than that predicted due to water-related infectious disease after mitigation.

**Table 9:** Current disease burden due to arsenic levels > 50 μg/L as a proportion of burden of disease due to other selected causes in Bangladesh.

		DALYs (%)	
Disease	Deaths (%)	Undiscounted	Discounted at 3%
All causes	0.9	0.3	0.4
Childhood-cluster diseases	34.2	8.1	14.7
Nutritional deficiencies	71.0	12.0	15.8
